# Supplementary material for: The risk of ischemic stroke significantly increases in individuals with blepharitis: A population-based study involving 424,161 patients
Source: PLoS One. 2023 Apr 27;18(4):e0284655. doi: 10.1371/journal.pone.0284655 (PMC10138796; doi:10.1371/journal.pone.0284655)
Supplement: Attachment — Submitted filename: PLoS One_reviewer 2_20221130.pdf [file pone.0284655.s001.pdf]

Response to reviewer 2's comment

Comment**:** It is a new idea. however, what about the type of infarction? Is it large vessel or small vessel disease. In addition, is it thrombosis or embolic stroke. And what do you recommend after getting these results about the relation between blepharitis and stroke?

Response: Thank you for your insightful comments. These are intriguing questions regarding the occluded arteries and type of ischemic stroke. But it was frustrating when we wanted to investigate the etiology further. Since this retrospective study defined study populations based on diagnostic codes, the information about location of culprit vessels and kind of ischemic stroke were not available in database. We concurred that these issues are crucial for validating the present findings. The origin of blood clots varies between thrombotic and embolic strokes. To minimize the potential bias, we adjusted the confounding factors such as non-valvular atrial fibrillation and venous thromboembolism in the analysis.

According to the findings of present study, we recommend early management of blepharitis. The risk of ischemic stroke persisted significantly higher in blepharitis cohorts than non-blepharitis cohorts for years according to the subgroup analysis. We hypothesized that chronic infection and inflammation played a crucial role in the relationship between blepharitis and ischemic stroke. Inflammatory cytokines may elevate immune response and pro-thrombotic states, hence stimulating thrombi development. We added additional detail in the Conclusions section.
