# Supplementary material for: The risk of ischemic stroke significantly increases in individuals with blepharitis: A population-based study involving 424,161 patients
Source: PLoS One. 2023 Apr 27;18(4):e0284655. doi: 10.1371/journal.pone.0284655 (PMC10138796; doi:10.1371/journal.pone.0284655)
Supplement: Attachment — Submitted filename: PONE-D-22-29971R1_reviewer 2_20230218.pdf [file pone.0284655.s002.pdf]

PONE-D-22-29971R1

Reviewer #2:

Thanks for addressing the comments. And one last comment on the word" causal " as it is written "casual" in discussion section please correct.

A: Thank you for your reminder and careful review, we correct this mistake.
